# Leveraging research infrastructure co-location to evaluate constraints on terrestrial carbon cycling in northern European forests

**DOI:** 10.1007/s13280-023-01930-4

**Published:** 2023-09-19

**Authors:** Martyn N. Futter, Thomas Dirnböck, Martin Forsius, Jaana K. Bäck, Nathalie Cools, Eugenio Diaz-Pines, Jan Dick, Veronika Gaube, Lauren M. Gillespie, Lars Högbom, Hjalmar Laudon, Michael Mirtl, Nikolaos Nikolaidis, Christian Poppe Terán, Ute Skiba, Harry Vereecken, Holger Villwock, James Weldon, Christoph Wohner, Syed Ashraful Alam

**Affiliations:** 1 Institutionen för vatten och miljö, Lennart Hjelms Väg 9, Box 7050, 75007 Uppsala, Sweden; 2 Vienna, Austria; 3 Finnish Environment Institute, Latokartanonkaari 11, 00790 Helsinki, Finland; 4 University of Helsinki, Helsinki, Finland; 5 Geraardsbergen, Belgium; 6 Institute of Soil Research, University of Natural Resources and Life Sciences, Peter-Jordan-Straße 82, 1190 Vienna, Austria; 7 Institute of Soil Research (IBF), Peter-Jordan-Straße 82, 1190 Vienna, Austria; 8 Skogforsk, Uppsala Science Park, 751 83 Uppsala, Sweden; 9 Department of Forest Ecology and Management, Swedish University of Agricultural Sciences, 901 83 Umeå, Sweden; 10 University of Crete, Chania, Greece; 11 IBG-3, Wilhelm-Johnen-Straße, 52428 Jülich, Germany; 12 UK Centre for Ecology & Hydrology, Bush Estate, Penicuik, EH26 0QB UK; 13 Agropshere Institute (IBG-3), Forschungszentrum Jülich Gmbh, 52425 Jülich, Germany; 14 Umweltbundesamt GmbH, Spittelauer Lände 5, 1090 Vienna, Austria; 15 Department of Forest Sciences, University of Helsinki, Latokartanonkaari 7, 00014 Helsinki, Finland

**Keywords:** Carbon: co-location, eLTER, Forest, Research infrastructure

## Abstract

Integrated long-term, in-situ observations are needed to document ongoing environmental change, to “ground-truth” remote sensing and model outputs and to predict future Earth system behaviour. The scientific and societal value of in-situ observations increases with site representativeness, temporal duration, number of parameters measured and comparability within and across sites. Research Infrastructures (RIs) can support harmonised, cross-site data collection, curation and publication. Integrating RI networks through site co-location and standardised observation methods can help answers three questions about the terrestrial carbon sink: (i) What are present and future carbon sequestration rates in northern European forests? (ii) How are these rates controlled? (iii) Why do the observed patterns exist? Here, we present a conceptual model for RI co-location and highlight potential insights into the terrestrial carbon sink achievable when long-term in-situ Earth observation sites participate in multiple RI networks (e.g., ICOS and eLTER). Finally, we offer recommendations to promote RI co-location.

## Introduction

Accurate assessments of the biogeochemical and societal controls on the contribution of forests to the terrestrial carbon sink are needed for robust climate change mitigation and adaptation policies in Europe and globally. Research Infrastructures (RIs) including the Integrated Carbon Observation System (ICOS; Heiskanen et al. 2022) and the Aerosol, Clouds and Trace Gases Research Infrastructure (ACTRIS; Wandinger et al. 2020) quantify forest: atmosphere carbon exchanges and their effects on the terrestrial carbon sink at a European scale. ICOS provides greenhouse gas (GHG) land: atmosphere exchange rate observations supported by high quality ancillary data (Heiskanen et al. 2022) as well as continental scale GHG patterns inferred from concentration inversions. ACTRIS offers standardised measurements of short-lived atmospheric climate forcers (Wandinger et al. 2020). Combining and contextualising measurements from the ICOS and ACTRIS atmospheric RIs with in-situ (ground-based) observations of relevant biogeochemical and socio-ecological drivers will shed light on processes—including human-nature interactions—controlling terrestrial carbon sequestration, why changes in terrestrial carbon stores are occurring and how these changes are related to other drivers including changes in land use, biodiversity and evolving societal priorities.

In-situ (site based) observations, remote sensing (Smith et al. 2020) and Earth System Models (ESMs; e.g., Baatz et al. 2018) all support the quantification and prediction of current and future terrestrial carbon sinks. Long-term and large-scale site-based observations are of special importance as they can be used to “ground-truth” remote sensing products. Combining long-term in-situ observations of carbon-related stocks, fluxes and model outputs can also lead to better ESM predictions (Baatz et al. 2021). Conclusions about the role of forests in terrestrial carbon storage drawn from remote sensing have been influential in the policy arena (e.g., Ceccherini et al. 2020), but can be inconsistent with in-situ observations (Palahi et al. 2021; Wernick et al. 2021). Using in-situ observations to corroborate remote sensing and ESM predictions process requires diverse yet comparable and standardised datasets derived from consistent observations. As datasets meeting these criteria are not yet readily available, there is an urgent need to harmonise and standardise data collection and management protocols in order to maximise the usability of site-based carbon storage and sequestration observations for both research and decision making.

Long-term observations made at single sites continue to play an important role in understanding human impacts on the environment. Atmospheric carbon dioxide measurements from Mauna Loa (Pales and Keeling 1965) provide some of the clearest evidence of temporal trends in atmospheric GHG concentrations while intensively studied Earth observation sites such as the Hubbard Brook Experimental Forest (Likens et al. 2021) give insight into ecosystem response to anthropogenic pressures. However, there is an increasing awareness that solving today’s environmental challenges requires more than single-site observations. Insights gained from large-scale and distributed RIs that coordinate, standardise and harmonise biophysical observations made at multiple and diverse sites (Loescher et al. 2022) complemented with observations of relevant contemporary socio-ecological changes and decision-making processes (Dick et al. 2018; Holzer et al. 2018) are needed. The scientific and societal value of long-term, site-based observations increases with temporal duration, the number of parameters measured, inter-operability with other data sources and timely availability of standardised, high quality data series. Through participation in two or more RIs (i.e., co-location), adoption of standardised observation protocols, uniform and well documented quality assurance and quality control (QA/QC) workflows and a commitment to FAIR (Findable, Accessible, Interoperable, Reusable; Wilkinson et al. 2016) data principles, the scientific and societal value of both sites and RIs are greater than the sum value of their individual elements.

Multiple European-scale RIs, including the Integrated European Long-Term Ecosystem, critical zone and socio-ecological Research Infrastructure (eLTER RI; Mirtl 2018), ICOS (Heiskanen et al. 2022), ACTRIS (Wandinger et al. 2020), the UNECE ICP Integrated Monitoring Programme (ICP-IM; Vuorenmaa et al. 2018) and ICP Forests (Ferretti 2021) deliver consistent, standardised multi-site data based on harmonised observing and QA/QC methodologies. This holistic “whole system” approach (Mirtl et al. 2018), where individual sites participate in multiple RIs, not only supports and facilitates scientific discovery but is needed if Europe is to achieve the goals of the Green Deal (EC 2019).

The aim of this paper is to highlight the potential for generating new insights into the terrestrial carbon sink based on observations made at long-term in-situ Earth observation sites participating in multiple RI networks. We present a conceptual model for RI co-location and use the Fennoscandic (northern European) forest as a case study to suggest possible improvements in understanding and managing the terrestrial carbon sink that can achieved with RI co-location. Finally, we offer recommendations for scientists, stakeholders and decision makers to better support the RI co-location process.

## Conceptual model for research infrastructure co-location

### Rationale

By participating in one or more RIs, individual sites gain access to a suite of services including data, access, computational and support services. Data services conforming to FAIR principles (Wilkinson et al. 2016) as well as standardised and quality controlled data products are provided through specific RI portals and ultimately through the European Open Science Cloud. Access services can be physical, remote and virtual for in-situ sampling and measurement campaigns as well as for off-site use of experimental facilities and scientific resources. Computational services can include virtual research environments, data analysis systems, visualization tools and modelling platforms. Support services include scientific workshop organization, education and training.

Holistic assessments of the terrestrial carbon sink require synthesis of information from environmental and societal domains. In the environmental (biophysical) domain, changes in above-ground carbon storage can be estimated in a number of ways including forest and soil inventory measurements (Neumann et al. 2016), upscaling of GHG flux measurements (Chi et al. 2019), satellite data assimilation (Smith et al. 2020) and modelling (Holmberg et al. 2019; Forsius et al. 2023). Such estimates must be complemented with measurements of belowground carbon storage and turnover as well as observations of relevant societal drivers. Obtaining the necessary cross-domain, multi-site measurements of societal drivers and the state of above- and below-ground carbon storage is easier when in-situ Earth observation sites implement standard monitoring and reporting protocols through multiple, co-located RIs.

### Model components

**Fig. 1:**
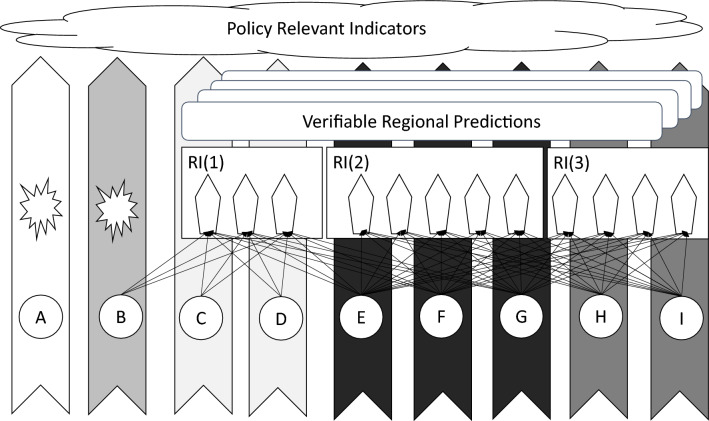
Components of a conceptual model for research infrastructure (RI) co-location. The model includes sites (sites A–I, represented as circles), long-term legacy observations (LLOs; stars), research infrastructures (RIs, rectangles), verifiable regional predictions (VRPs; rectangles with rounded corners), standard observation protocols (SOPs; pentagonal arrowheads) and policy relevant indicators (PRIs; cloud). Arrows connecting sites to an SOP indicate that the site makes observations using a standardised protocol suitable for generation of a VRP. Each SOP is owned by an RI. The ability of a site to contribute to PRIs is indicated by the darkness of the vertical arrows connecting sites and PRIs with darker arrows indicating stronger contributions (e.g., sites E, F and G make a stronger contribution than sites C and D). There are three RIs in the figure (RI(1)–RI(3)). RI(1) makes observations using three SOPS, RI(2) has five and RI(3) has four. Only observations made using SOPs can contribute to the development of VRPs. Site A only collects LLOs. This, it has the least ability to support PRIs and is not able to contribute to the generation of VRPs. Sites C and D participate in a single RI (RI(1)) and make observations using SOPs developed by that RI. Site B can make a more effective contribution to PRIs as it has both LLOs and participates in an RI where it makes observations using SOPs. As sites H and I co-locate RI(2) and RI(3), they can contribute to the development of VRPs. Furthermore, they have a greater ability to contribute to PRI development than sites that do not have co-located RIs. Sites E, F and G can make the strongest contribution to PRI development as they make observations using SOPs for all three RIs

A conceptual model can help to understand benefits and potential drawbacks of RI co-location. The model should include the following components: in-situ observing sites, research infrastructures, standardised observing protocols, long-term legacy observations, verifiable regional predictions and policy relevant indicators (Fig. 1).

### Sites

A site is a definable geographical area where in-situ observations are made of the natural and/or human environment that support research and societal decision-making. Site personnel make in-situ observations and manage data. Typically, sites are operated by a single public sector entity (e.g., universities, government agencies or research institutes). Sites are either owned by the managing entity or secured under long-term agreement. Site operations are usually funded through a range of short- or long-term financial instruments including competitive grants, service contracts, internal institute resources and various national and European programmes (Table 1).

**Table 1:** Acronyms used

Acronym	Definition
ACTRIS	Aerosol, Clouds and Trace Gases Research Infrastructure
CLRTAP	Convention on Long-range Transboundary Air Pollution
DEIMS	Dynamic Ecological Information Management System
DPSIR	Driver Pressure State Impact Response Framework
eLTER	European Long-Term Ecosystem, critical zone and socio-ecological systems Research
ESM	Earth System Model
EU	European Union
FAIR	Findable, Accessible, Interoperable, Reusable
GHG	Greenhouse Gas
ICOS	Integrated Carbon Observation System
ICP	UNECE International Cooperative Programme
ICP-IM	UNECE ICP Integrated Monitoring
LSCSS	Landscape Scale Carbon Storage and Sequestration
LLO	Long-term Legacy Observation
ILTER	International Long-term Ecological Research
LTSER	Long-term Socio-ecological Research
LULCC	Land Use/Land Cover Change
NBP	Net Biome Productivity
NEON	US National Ecological Observatory Network
NEP	Net Ecosystem Productivity
PRI	Policy-Relevant Indicator
QA/QC	Quality assurance/quality control
RI	Research Infrastructure
SOP	Standard Observing Protocol
TERENO	German Terrestrial Environmental Observatories
UNECE	United Nations Economic Commission for Europe
US-LTER	US National Science Foundation Long Term Ecological Research
VRP	Verifiable Regional Prediction

Sites do not necessarily have sharp geographical boundaries but can be geo-located using a centroid, representative coordinates or bounding polygons. Usually, sites have one or more observing locations (Wohner et al 2022). Observations can be made using instruments (e.g., weirs, flux towers), surveys (e.g., vegetation, birds, etc.), collection of material (e.g., sampling biota or soil), document analysis (e.g. land ownership records) or human contact (e.g., interviews, surveys).

Sites can participate in zero or more RIs. Depending on available resources and awareness levels, site data are managed in a more or less structured manner, ranging from effectively undocumented spreadsheets on individual researcher’s computers through robust, centralised IT systems with all necessary metadata (e.g., the ICOS Carbon Portal, Heiskanen et al. 2022). Data management is easier to professionalise when observing and reporting methods are harmonised, as is the case at sites participating in RIs. Such sites are typically more committed to FAIR principles and third party data access. Criteria for third party access and use of data can range from none through complicated and restrictive licenses. Increasing pressure from the scientific community, funding bodies and the European Commission to ensure data are compliant with FAIR principles (Wilkinson et al. 2016) is gradually improving data access, as is RI membership and the associated demands for standardised data management. Online catalogues (e.g., DEIMS; Wohner et al. 2019) are important sources of site metadata and there is increasing recognition of the need to develop the necessary IT infrastructure for sharing data between RIs (Huber et al. 2021). Crediting data authors using internationally recognised and persistent identifiers can also improve data access (Rennie et al. 2021).

### Research infrastructures

Research Infrastructures (RIs) are “umbrella” organisations that coordinate observations made at multiple sites. RIs promote the use of standardised monitoring and reporting protocols and offer centralised data services. The European environmental RI landscape includes first, second and third generation infrastructures making observations that can contribute to an understanding of the terrestrial carbon sink (Table 2). National forest inventories, some of which have been in continuous operation for more than a century (McRoberts et al. 2010), are probably the most relevant first generation RI for landscape-scale carbon assessments. However, it is not always straightforward to use these data to quantify terrestrial carbon stocks (Guintoli et al. 2020) as forest inventory RI reporting is focused on assessments of above-ground wood production, not carbon accounting and they fail to adequately consider below-ground carbon storage.

**Table 2:** Properties of first, second and third generation European environmental research infrastructures (RIs) relevant to assessments of terrestrial carbon sequestration and storage. Extensive RIs typically make a limited number of observations at a large number of sites while intensive RIs make a large number of observations at a small number of sites

	Generation		
	First	Second	Third
Example	National Forest Inventories	UNCE ICP-IM and ICP Forests	ACTRIS, ICOS
FAIR principles compliance	Low to Moderate	Moderate	High
Extensive/intensive	Extensive	Intensive and Extensive	Intensive
Standardised observing protocols	Yes	Yes	Yes
Compatibility of protocols across RIs	Low	Low to Moderate	Moderate to High
Scope	National	International	Continental

Second generation RIs making observations relevant for understanding the terrestrial carbon sink include the UNECE ICP-IM (Vuorenmaa et al. 2018) and ICP Forests (Ferretti 2021) programmes. These RIs were established following a top-down process under the Convention on Long Range Transport of Air Pollution (CLRTAP; Wettestad 1997). The ICP programmes collect policy-relevant environmental data at sites across Europe using Standardised Observing Protocols (SOPs; described below). In some cases, RIs have been making observations for almost 50 years. These long-term, standardised observations have contributed to the evidence base for emissions reduction agreements to mitigate acidification (Wettestad 1997; Dirnböck et al. 2018; Grennfelt et al. 2020) as well as for assessments of forest health. For example, Penuelas et al. (2020) used ICP Forests data to highlight the widespread decline in foliar nutrient status of European forests and to raise a warning flag about possible loss of resilience in terrestrial carbon sequestration that would increase the uncertainty of carbon budget estimates and resulting climate change mitigation policies.

The value of first and second generation RIs should not be underestimated as they have the longest observation time series, often dating back to the first half of the twentieth century. However, these RIs are not used to the fullest possible extent because their data holdings can be difficult to access. This is a legacy of their original purpose; first generation RIs originally supported national government oversight of natural resources, and second generation RIs are closely linked to CLRTAP reporting requirements. While some data produced by these RIs are open or available (e.g., by request), the historical focus of first and second generation RIs on generating data for internal use has meant that they did not prioritise the application of FAIR or other third party access principles. Today, there are encouraging moves towards greater transparency and open data access, especially amongst second generation RIs focused on evaluation of the effectiveness of international conventions and protocols.

The US LTER infrastructure was one of the first third generation RIs (Keller et al. 2008). In many ways, it provided a blueprint and inspiration for subsequent RIs including TERENO (Zacharias et al. 2011), NEON (Keller et al. 2008) and the ILTER network of networks (Mirtl et al. 2018; Dirnböck et al. 2019). The success of these RIs was in large part due to secure and sustained funding needed to support high quality observations, long-term data collection and information management. Third generation RIs are characterised by significant investments in IT infrastructure, documented QA/QC protocols, a commitment to the use of SOPs and timely and openly available data. Because of these characteristics, ICOS (Heiskanen et al. 2022) and other third generation RIs are widely used to develop the Verifiable Regional Predictions (VRPs, described below) needed for high-impact, policy-relevant science (e.g., Migliavacca et al. 2021).

In addition to the knowledge that can be gained from single RI observations, combining observations from multiple generations of RIs can give new insights into the terrestrial carbon sink. Cabon et al. (2022) combined first generation long-term growth measurements based on tree rings with third generation eddy covariance measurements to better constrain estimates of terrestrial carbon sequestration. These advances based on observations from multiple co-located RIs are laying the groundwork for the next generation of syntheses that connect site biogeochemistry to biodiversity and socio-ecology.

### Standard observation protocols (SOPs)

Standard Observation Protocols (SOPs) are publicly accessible descriptions of reproducible and affordable mechanisms for characterizing and reporting environmental system states or processes. A SOP is owned and maintained by an RI (e.g., ICOS protocols for flux measurement, ICP-Forests protocols for vegetation monitoring) or third party (e.g., World Meteorological Organization protocols for precipitation monitoring). A SOP must be sufficiently well documented to allow cross-site implementation, integration and comparison, e.g., observations made using the ICP-Forests^1^ and ICOS (Gielen et al. 2018) vegetation monitoring protocols can be compared with each other. One main benefit of using SOPs is that they allow sites to collect observations in a consistent and harmonised manner. Consistent and harmonised observing protocols and standardised QA/QC procedures greatly simplify the synthesis of data from multiple sites and the production of VRPs.

### Long-term legacy observations (LLOs)

Many sites have long time series of environmental observations, or Long-term Legacy Observations (LLOs). Protocols for LLOs are typically owned at the site level (e.g., each site will have their own protocols for observing, QA/QC routines and data storage protocols). Metadata characterizing LLO protocols may be more or less readily available, potentially hindering implementation across sites as well as making it difficult for an individual site to share data or to make cross-site comparisons. One strength of many LLO series is continuity of measuring technique, which greatly simplifies detection and attribution of signals in the data. The main perceived drawback of LLOs is that they are difficult to compare across sites.

The insights about long-term environmental change that can be extracted from analysis of LLOs are an important part of the evidence base for Policy Relevant Indicators (PRIs; described below). However, conclusions derived from LLOs lack explicit regional context. National funders, site managers and RIs should prioritise efforts to develop inter-calibration tools for LLOs from observing sites across Europe in a seamless and harmonised manner as robust methods for translation across observing protocols are vital for informed environmental policy. To give one example, the EU Water Framework Directive has a goal of achieving good ecological status in surface waters across Europe. Status assessments are based on both standardised observations and LLOs that can differ between countries. Because different countries had invested heavily in different observing strategies, there was no realistic possibility of new, pan-European SOPs. This institutional bottleneck led to significant investment of scientific effort to inter-calibrate the different LLOs used across Europe for water quality monitoring (Poikane et al. 2015).

### Verifiable regional predictions (VRPs)

Verifiable Regional Predictions (VRPs) are one of the most important computational services currently provided by eLTER and other RIs as they contribute to the evidence base for policies to address current and future environmental challenges. VRPs are based on measurements made using SOPs at sites participating in a RI. A key feature of VRPs is that they leverage site-based, geographically discontinuous point measurements to produce indicators (e.g., Critical Loads) that have seamless spatial and temporal coverage. Inputs to VRPs may include modelling (e.g., Xu et al. 2021) and remote sensing (e.g., Ceccherini et al. 2020) but must be “ground truthed” by in-situ measurements (e.g., Pongratz et al. 2021). The spatial distributions of predictions and their associated uncertainty in VRPs derived from observations at existing sites may highlight the need for new observing sites (e.g., Wohner et al. 2021) or co-location of additional RIs at existing observing sites (as is being pursued by ACTRIS Sweden).

### Policy relevant indicators (PRIs)

Policy Relevant Indicators (PRIs)—new, actionable insight into system behaviour—can be derived from single site LLOs or VRPs created from multi-site observations collected using SOPs. A PRI is not the same as a policy action. PRIs provide the evidence base that decision makers can use in policy formulation as a precursor to action. The research community develops PRIs but policy decisions are ultimately made by elected decision makers and civil society.

Connections between VRPs and PRIs must be strengthened if the site-based research community is to respond and contribute to implementing the European Green Deal (EC 2019) and other societal challenges. Facilitating societal change based on the new knowledge gained from VRPs will require an appropriate and implementable communication strategy. The Green Deal identifies a number of possibilities including simple “traffic light” approaches where indicators are colour coded red, yellow or green depending on severity (Futter et al. 2016). Another approach would be to use the strategy exemplified by Högbom et al. (2021) where scientific synthesis supported targeted communication with EU decision makers.

Both spatially extensive multi-site RI-based (i.e., VRPs) and single-site long-term (i.e. LLO) indicators are needed to support the policy process. LLOs can be used to identify background or reference conditions, as well as being an indicator of progress towards environmental goals. National decision makers, RIs and the site-based Earth observation community must all collaborate to ensure that the wealth of existing observations that exist in site data archives are incorporated into the provision of actionable RPOs.

### Other aspects of RI co-location

While RI co-location has multiple benefits, there can be drawbacks. RI membership can be seen as an unnecessary expense at the site and national levels. Sites may also struggle to implement SOPs and may be reluctant to abandon LLO protocols. Nationally, funding agencies may feel pressured to abandon sites that do not participate in RIs. Also, the push towards FAIR and open data may not always be supported by data providers. Recognising the perceived drawbacks of RI co-location and addressing them in an open and constructive manner will contribute to stronger European science that is better able to address future environmental challenges.

Physical co-location (site interoperability), where two or more RIs make observations at the same or nearby geographical coordinates, is a necessary condition for interoperability. However, the importance of virtual co-location (data interoperability; Wohner et al. 2020; Huber et al. 2021), should not be neglected. Standardised QA/QC protocols and a commitment to FAIR data principles greatly increase the value of multi-site observations to scientists and other end-users.

## The terrestrial carbon sink: What, how and why

The terrestrial carbon sink is an important part of the EU Green Deal strategy for reaching net carbon neutrality (EC 2021). Maintaining or increasing rates of forest carbon sequestration and the use of forest products to replace fossil carbon intensive materials both contribute to achieving this goal (Forest Europe 2020). However, there is increasing evidence that the terrestrial carbon sink is declining and that European forests may be approaching saturation (Nabuurs et al. 2013). This along with, e.g., changes in forest element cycling (Penuelas et al. 2020), concerns about assumptions behind models used to estimate future forest carbon storage as well as an understanding of the way land managers make decisions (Guintoli et al. 2020) all highlight the need for credible long-term, standardised observations of carbon sequestration and storage, improved scientific understanding of mechanisms and processes controlling the terrestrial carbon sink, and a greater awareness of the relevant societal driving factors.

Assessments of the terrestrial carbon sink require standardised observations of carbon pools and fluxes over time. Change in storage over time equals the average sink over the period of interest, i.e. the rate of change in storage over time gives the sink. Data from multiple RIs (e.g., ACTRIS, ICOS, eLTER, etc.) collected using SOPs are needed to quantify biological and non-biological components of the carbon sink. The biological carbon sink is equal to Net Ecosystem Productivity (NEP). The total carbon sink is given by Net Biome Productivity (NBP). This includes the biological sink as well as non-respiratory processes removing carbon, e.g., lateral fluxes by water, fires and harvesting.

**Fig. 2:**
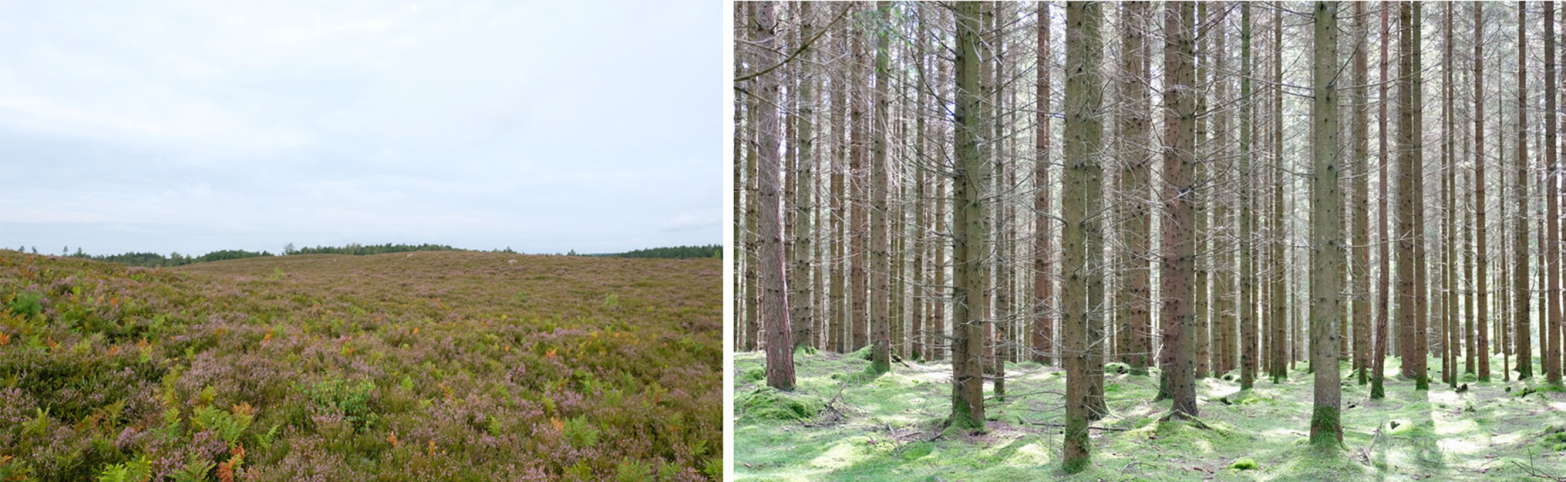
Legacies of past land management: two geographically proximate Swedish landscapes, one heathland. (left) and one forest (right) The difference in carbon storage in these two landscapes with very similar biogeophysical conditions is a legacy of past management. Photos © Lars Högbom

The wide range of climate and light conditions across Europe as well as the legacy of past land management all lead to a strong differentiation in the potential carbon sink. The forest carbon sink can vary depending on management intensity, ranging from protected and/or unmanaged areas with high storage but low sequestration to intensive production landscapes with lower total storage but higher rates of biological sequestration. Terrestrial carbon storage is constrained by the legacy of past societal decision-making (Fig. 2) and past land management strongly influences future terrestrial carbon storage potential (Thom et al. 2018). This highlights the need for a better understanding of present and past societal decision-making processes. Long-Term Socio-Ecological Research (LTSER) Platforms synthesise long-term historical data extracted from archival and statistical sources to reconstruct socio-economic changes and link them to current management practices (e.g., Angelstam et al. 2019). Including LTSER platforms in the RI co-location process will help to clarify the relative importance of societal and biophysical factors controlling the terrestrial carbon sink.

### RI co-location for better understanding the forest carbon sink in northern Europe

First, second and third generation RIs as well as remote sensing and climate reanalysis products are all important data sources for understanding, managing and communicating the northern European forest carbon sink. Long-term measurements from first generation RIs including national forest inventories (Neumann et al. 2016), second generation RIs such as the ICP programmes (Holmberg et al. 2018; Penuelas et al. 2020) as well as third generation RIs including ICOS (Heiskanen et al. 2022), ACTRIS and eLTER (Mirtl et al. 2018) can all play a role in quantifying terrestrial carbon dynamics.

Co-located RIs where multiple, regionally representative sites observe the environment using standardised protocols can provide the necessary evidence base to support decision making, especially when sites with co-located RIs are augmented with interdisciplinary LTSER Platforms and approaches that focus on emergent socio-ecological systems. RI co-location at LTSER Platforms can connect observations of carbon cycling to resource use, governance and communication. Connecting sites to LTSER platforms can also facilitate consideration of patterns and processes across multiple spatial and temporal scales and facilitate the transition to participation-oriented sustainability science.

RI communities are increasingly recognising the benefits of co-location for improved understanding of the Earth system. Although ICOS Ecosystem Sites focus primarily on flux measurements relevant for ecosystem carbon cycling, these sites also make observations relevant for understanding biophysical and socio-ecological processes that influence GHG fluxes (Saunders et al. 2018). These ancillary observations are collected using SOPs and can (in principle) be translated for comparability with LLOs or measurements based on other SOPs. Having greater clarity about the additional observations needed to support VRPs would increase the value of GHG flux measurements. Specifically, a suite of harmonised biogeochemical, biodiversity and socio-ecological observations made using SOPs with robust QA/QC checks across a broad range of well-supported sites and LTSER Platforms are needed for an adequate understanding of the terrestrial carbon sink.

The eLTER RI offers such a suite of observations. The RI takes a “whole-system” approach to social and ecosystem processes in four focus areas: biodiversity dynamics, climate change, biogeochemistry and socio-ecological systems (Mirtl et al. 2018). With its strong reliance on landscape-scale LTSER Platforms, the eLTER RI measures socio-economic drivers, environmental pressures related to substance release (including GHGs) and land use/land cover change (LULCC), state indicators documenting ecosystem impacts, pressures that negatively impact desired ecosystem functioning, and societal response to environmental change.

A holistic understanding of carbon dynamics in northern European forests requires multiple forms of knowledge. These include: current and future societal perspectives on the bioeconomy (Rakovic et al. 2020); robust assessment of GHG fluxes (e.g., Chi et al. 2019); quantification of ecosystem pressures (Vuorenmaa et al.^2018^); new insights into controls on ecosystem service delivery (Holmberg et al. 2019; Laudon et al. 2021); and possible policy responses (Venäläinen et al. 2020; Högbom et al 2021). Synthesizing new knowledge across each of these areas is a prerequisite for meeting the ambitious demands of the European Green Deal.

Multiple RIs co-located at sites across Europe can help to answer three main questions about the terrestrial carbon sink:

(i) What are the current and cumulative rates of terrestrial carbon sequestration and storage?
(ii) How are carbon sequestration rates mediated by other ecosystem processes?
(iii) Why do the observed patterns of terrestrial carbon sequestration and storage occur?

We explore these three questions using the northern European (Fennoscandic) forest as a case study. Understanding the Fennoscandic forest is relevant both from European and global perspectives as it is a “living lab” offering insights about conditions in northern forests globally (see Venäläinen et al. 2020). Forests cover 35% of the European land mass and more than two thirds of the European forest area is located in the boreal and temperate ecozones (Forest Europe 2020). The Fennoscandic forest is relatively homogeneous due to a long history of management including timber production and fire suppression with relatively little set aside for nature conservation or other values (Högbom et al. 2021).

### What are current and cumulative rates of carbon storage and sequestration in northern European forests?

Policy relevant indicators of past and present carbon sinks in the northern European forest can be obtained from observations collected by sites participating in first, second and third generation RIs. The value of these indicators for policy support increases with RI co-location (Fig. 1). Co-location facilitates the use of SOPs from multiple RIs for making Earth system observations needed for carbon science. The ICOS ecosystem RI “supersites” in Sweden (Laudon et al. 2021) and Finland (Hari et al. 2016) are flagships for carbon science. Through participation in the ICOS and eLTER RIs, they make all necessary observations for quantifying changes in the terrestrial carbon sink.

Co-location of the ACTRIS RI at sites participating in ICOS and eLTER will further improve our ability to understand atmospheric controls on forest carbon cycling. ACTRIS observations of short-lived atmospheric components are augmented by more than 100 years of measurements from Swedish sites that currently host ICOS infrastructure (see Malmström 1923). New process understanding could be obtained if these legacy measurements can be combined with insights from, e.g., national forest inventories (Laudon et al. 2021), the UNECE ICP programmes (Jungqvist et al. 2014) and next generation tools for landscape-scale analysis (Laudon et al. 2022).

An application of the dynamic forest carbon balance PREBAS model (Minunno et al. 2019) provides a relevant example of data aggregation and extrapolation in this context. PREBAS was initially calibrated to intensive data collected at ICOS sites, and then calibrated to data sets from the Finnish national forest inventory to provide national-scale VRPs of changes in GHG fluxes and carbon storage in Finland under different management and climate scenarios (Minunno et al. 2019; Forsius et al. 2023; Mäkelä et al. 2023). Cabon et al. (2022) highlighted the possibility of new insights when RIs are co-located. They assessed source-sink controls on forest carbon cycling using observations from a combination of first and third generation RIs. They combined dendrochronology data for estimating tree growth with eddy covariance Gross Primary Productivity estimates to evaluate and quantify the coupling between carbon sequestration and growth increment. Their findings showed significant disconnections between atmospheric fluxes and long-term carbon storage, highlighting the need for multiple lines of evidence for robust assessments of net carbon sinks.

### How is forest carbon storage mediated by other biogeochemical and physical processes?

Rates of forest carbon sequestration depend on biological processes (e.g., Holmberg et al. 2019). Clearly, the dominant vegetation type (e.g., forest or heathland) is an important factor. Physiologic controls on carbon allocation (Cabon et al. 2022), resilience to drought (Kupec et al. 2021) and inherent growth rates all vary across tree species and varieties. The dominant vegetation type cannot be considered in isolation. Other components of the ecosystem are also relevant. Nutrient cycling, and hence forest carbon storage depends on mycorrhizal associations (Mäkelä et al. 2022). Forest resilience to wind throw is dependent on herbivory, which is an important control (Leroux et al. 2020) with moose and reindeer being especially important in the region.

Climate change will lead to warmer soils in northern Europe (Jungqvist et al. 2014). Lateral carbon fluxes are also likely to increase (Oni et al. 2015). Changing nitrogen (N) cycling in northern forests (Sponseller et al. 2016) will also affect terrestrial carbon cycling. This highlights the need to incorporate N limitation and N feedback effects in ecosystem and global models used in climate change assessments (Norby et al. 2010). Furthermore, there is a need to include possible consequences of phosphorus, micronutrient and water limitations on the forest carbon sink. While more robust observations and a better understanding of biogeochemical processes will contribute to the development of VRPs about how carbon cycles through the landscape, these must be complemented by new insights into the societal factors explaining why observed patterns exist.

### Why do the observed patterns in forest carbon sinks exist?

All European landscapes are a legacy of past societal decisions (Emanuelsson 2009). A complete understanding of the terrestrial carbon sink requires knowledge about people living and working in the forest and the decisions they make. Much of the Fennoscandic forest is intensively managed for timber production and increasingly for other ecosystem services (Högbom et al. 2021) and in both Sweden (Petersson et al. 2022) and Finland (Forsius et al. 2023) the forestry sector is an important player in achieving carbon goals.

LTSER Platforms (Holzer et al. 2018; Angelstam et al. 2019) focus on addressing sustainability challenges through better integration of social perspectives into long-term ecological research. These platforms encompass LTER Sites, but also include the broader geographic area that contains them, along with cultural, administrative, historic, economic and other social dimensions of the region. Such information is vital to understand the temporal trends in forest carbon. Platform integration is another important issue. In a globalised world, isolated landscape-scale studies would fail to address important issues. National and supra-national levels must be considered as well as cooperation between LTSER Platforms to conduct cross-platform, place-based comparative studies (Geist and Lambin 2002).

Differing stakeholder priorities lead to multiple conflicts and trade-offs in northern European forests and these societal factors can influence carbon cycling. In a study of stakeholder priorities in northern European forests, Högbom et al. (2021) identify a “trilemma” of timber production, climate services and biodiversity conservation. They suggested that prioritizing one dimension can lead to trade-offs in the other two, e.g., conventional forest management for timber production prioritises younger stands at the expense of old growth biodiversity and long term forest carbon storage. Transparent and open communication with all stakeholders and decision makers will not eliminate trade-offs but could make them more acceptable if they can be seen as a co-created solution instead of an external imposition. The study of communication and knowledge formation during long-term interactions about the goals of forest management can contribute to the co-creation of acceptable trade-offs by assessing transformations, clarifying the role of actors in their network, and highlighting past and future governance structures.

## Conclusions

Achieving European climate policy goals will require more than just political and societal will. It will require effective participation by scientists in the policy process (Mubareka et al. 2022), a renewed commitment by national funders and more active outreach and engagement by the site-based research community. National funders must take a more inclusive attitude to supporting environmental monitoring. Formal cooperation agreements such as the ones eLTER has made with CLRTAP and ICOS must become more common and easier to achieve. Such agreements set the stage for concrete modes of cooperation including uptake and use of standardised observation protocols.

Physical co-location, as is being pursued by ACTRIS and ICOS in Sweden, offers clear benefits for building the knowledge base needed for a sustainable future, especially when supported by virtual co-location of information systems. From the perspective of an individual in-situ Earth observation site, RI co-location offers the potential for a more holistic understanding of ecosystem structure, function and interactions, more cost-efficient operations (both in terms of personnel and instrumentation) as well as the harmonised and interoperable data needed to address societal challenges.

Physical co-location of multiple RIs is needed to address tomorrow’s environmental challenges, and the scientific community must continue to promote active collaboration. While physical co-location is necessary, it is not sufficient. Information sharing and the IT systems to support sharing must also be prioritised. Commitments to FAIR data principles (Wilkinson et al. 2016) must be strengthened and rewarded by both the scientific community and funding agencies. Significant new investments are needed to ensure that long-term legacy observations are not lost and can be used in conjunction with new measurements made using standardised protocols.

The eLTER vision must be expanded. Existing LTSER Platforms in protected areas must be supported, but they should be complemented with the creation of new platforms encompassing sites in production landscapes and better efforts to co-locate with new and existing sites participating in ACTRIS, ICOS, and other European RIs.

## Footnotes

1 (http://icp-forests.net/page/icp-forests-manual, last accessed 30/6/2023).
