# Publisher Correction: Leveraging research infrastructure co-location to evaluate constraints on terrestrial carbon cycling in northern European forests

**DOI:** 10.1007/s13280-023-01941-1

**Published:** 2023-10-03

**Authors:** Martyn N. Futter, Thomas Dirnböck, Martin Forsius, Jaana K. Bäck, Nathalie Cools, Eugenio Diaz-Pines, Jan Dick, Veronika Gaube, Lauren M. Gillespie, Lars Högbom, Hjalmar Laudon, Michael Mirtl, Nikolaos Nikolaidis, Christian Poppe Terán, Ute Skiba, Harry Vereecken, Holger Villwock, James Weldon, Christoph Wohner, Syed Ashraful Alam

**Affiliations:** 1 Institutionen för vatten och miljö, Lennart Hjelms Väg 9, Box 7050, 75007 Uppsala, Sweden; 2 Vienna, Austria; 3 Finnish Environment Institute, Latokartanonkaari 11, 00790 Helsinki, Finland; 4 University of Helsinki, Helsinki, Finland; 5 Geraardsbergen, Belgium; 6 Institute of Soil Research, University of Natural Resources and Life Sciences, Peter-Jordan-Straße 82, 1190 Vienna, Austria; 7 Institute of Soil Research (IBF), Peter-Jordan-Straße 82, 1190 Vienna, Austria; 8 Skogforsk, Uppsala Science Park, 751 83 Uppsala, Sweden; 9 Department of Forest Ecology and Management, Swedish University of Agricultural Sciences, 901 83 Umeå, Sweden; 10 University of Crete, Chania, Greece; 11 IBG-3, Wilhelm-Johnen-Straße, 52428 Jülich, Germany; 12 UK Centre for Ecology & Hydrology, Bush Estate, Penicuik, EH26 0QB UK; 13 Agropshere Institute (IBG-3), Forschungszentrum Jülich Gmbh, 52425 Jülich, Germany; 14 Umweltbundesamt GmbH, Spittelauer Lände 5, 1090 Vienna, Austria; 15 Department of Forest Sciences, University of Helsinki, Latokartanonkaari 7, 00014 Helsinki, Finland

**Publisher Correction to: Ambio** 10.1007/s13280-023-01930-4

In the original publication of article, a typo was introduced in the co-author name during the creation of the article proof. The author's name "Holger Villwock" was incorrectly written as "Holger Villock".

The original article has been corrected.

